# C-Kit^+^ cells can modulate asthmatic condition via differentiation into pneumocyte-like cells and alteration of inflammatory responses via ERK/NF-ƙB pathway

**DOI:** 10.22038/IJBMS.2021.59946.13293

**Published:** 2022-01

**Authors:** Fatemeh Mirershadi, Mahdi Ahmadi, Reza Rahbarghazi, Hossein Heiran, Rana Keyhanmanesh

**Affiliations:** 1 Department of Physiology, Faculty of Medicine, Tabriz University of Medical Sciences, Tabriz, Iran; 2 Department of Physiology, Ardabil Branch, Islamic Azad University, Ardabil, Iran; 3 Student Research Committee, Tabriz University of Medical Sciences, Tabriz, Iran; 4 Tuberculosis and Lung Disease Research Center, Tabriz University of Medical Sciences, Tabriz, Iran; 5 Stem Cell Research Center, Tabriz University of Medical Sciences, Tabriz, Iran; 6 Department of Applied Cell Sciences, Faculty of Advanced Medical Sciences, Tabriz University of Medical Sciences, Tabriz, Iran; 7 Drug Applied Research Center, Tabriz University of Medical Sciences, Tabriz, Iran

**Keywords:** Asthma, C-kit cells, CD4+/CD8+, Differentiation, NF-ƙB, p-ERK/ERK

## Abstract

**Objective(s):**

The exact role of the progenitor cell types in the dynamic healing of asthmatic lungs is lacking. This investigation was proposed to evaluate the effect of intratracheally administered rat bone marrow-derived c-kit^+^ cells on ovalbumin-induced sensitized male rats.

**Materials and Methods:**

Forty rats were randomly divided into 4 groups; healthy rats received phosphate-buffered saline (PBS) (C); sensitized rats received PBS (S); PBS containing C-kitˉ cells (S+C-kit^-^); and PBS containing C-kit^+^ cells (S+C-kit^+^). After two weeks, circulatory CD4^+^/CD8^+^ T-cell counts and pulmonary ERK/NF-ƙB signaling pathway as well as the probability of cellular differentiation were assessed.

**Results:**

The results showed that transplanted C-Kit^+^ cells were engrafted into pulmonary tissue and differentiated into epithelial cells. C-Kit^+^ cells could increase the number of CD4^+^ cells in comparison with the S group (*P*<0.001); however, they diminished the level of CD8^+^ cells (*P*<0.01). Moreover, data demonstrated increased p-ERK/ERK ratio (*P*<0.001) and NF-ƙB level (*P*<0.05) in sensitized rats compared with the C group. The administration of C-kit^+^, but not C-Kit^-^, decreased p-ERK/ERK ratio and NF-ƙB level compared with those of the S group (*P*<0.05).

**Conclusion:**

The study revealed that C-Kit^+^ cells engrafted into pulmonary tissue reduced the NF-ƙB protein level and diminished p-ERK/ERK ratio, leading to suppression of inflammatory response in asthmatic lungs.

## Introduction

Asthma is a complex pathological condition with an allergic response in the broncho-pulmonary segment that threatens human health (1, 2). It was suggested that asthma coincides with the chronic inflammatory response which is caused by the activity of CD4+ T lymphocytes, namely, type 2 T helper (Th2)-immune response (3, 4). Upon stimulation of Th2 cells, arrays of cytokines are released into the asthmatic niche (5, 6). As a correlate, the dynamic balance of Th1/Th2 cells and significant production of interleukins (ILs) such as IL-4, -5 and -13 are thought to be involved in the recruitment of different subsets of inflammatory cells and subsequent airway hyper-responsiveness (7, 8). Moreover, the activity of antigen-primed CD8+ T cells is indispensable for the development of allergen-induced airway inflammation and hyper-responsiveness during the progression of asthma (9). Many studies have described the critical role of mitogen-activated protein kinase (MAPK) and extracellular signal-regulated kinase/Nuclear factor-κB (ERK/NF-κB) signaling pathways in the progression/suppression of allergic asthma responses (10, 11). NF-κB is an important transcription factor contributing to the expression of various pro-inflammatory genes. Increased ERK/NF-κB activity has been documented in airway epithelial cells and alveolar macrophages of asthmatic patients (12). Inhalation of corticosteroids in combination with long-acting β2agonists or leukotriene modulators can commonly be administrated to relieve asthmatic symptoms (13). Because of the possibility of serious side effects (14, 15), new therapeutic approaches are needed. 

Along with conventional therapeutic strategies, regenerative medicine is substantial to treat diseases with no accessible or effective treatments (16). For instance, transplantation of different cell lineages alone or in combination with growth factors and cytokines can help the host tissue to restore, but not completely, physiological function (17, 18). Among several cells that exist inside tissues, stem cells are expected to possess the unique regenerative potential for dealing with several diseases (19). C-kit^+^ cells are one of the stem cell subgroups with several regenerative capacities. It has been indicated that activation of the C-kit, a stem cell factor (SCF) receptor, can contribute to indispensable functions of effectors and signaling proteins inside human cells (20). For instance, SCF/C-Kit signaling has been delineated to have an essential role in the modulation of phenotype acquisition of microglia and intensity of immune responses (21-24). Transplantation of human C-Kit^+^/SSEA4^−^ retinal progenitor cells has been shown to diminish the frustration of microglia and inflammation intensity in rats and mice with improved vision rate (25). In line with this statement, Ellison and colleagues showed that the adult heart encompasses numerous endogenous c-kit^+ ^cardiac stem cells that could repair the injured myocardium during several pathologies (26). These cells exhibit the typical characteristics of stem cells such as clonogenicity, self-renewal, with prominent capacity committed into cardiomyocytes, smooth muscle, and endothelial cells (27, 28). To the best of our knowledge, few reports are highlighting the c-Kit^+^ progenitor cells during different inflammatory conditions. Here, we proposed to evaluate the effect of intratracheally administered rat bone marrow-derived c-kit^+^ cells on the ovalbumin-induced sensitized model of male rats. Two weeks after implementation of these cells, circulatory CD4^+^ and CD8^+^ T-cell counts and pulmonary ERK/NF-κB signaling pathway were assessed. In addition, the probability of differentiation of these cells was appraised.

## Materials and Methods

***Animal ethics***

All experimental procedures followed guidelines of the care and use of laboratory animals (NIH Publication No. 85-23, revised 1996) and were confirmed by the Animal Care Committee of Tabriz University of Medical Sciences (No: IR.TBZMED.VCR.REC.1397.404).

***Experimental groups***

In this study, fifty male rats (weighing 200–250 g, 8–9 weeks old) were enrolled in the study. These animals were purchased from the animal house of Tabriz University of Medical Sciences and kept in standard condition and permitted to access chewing food and water ad libitum. Fourteen days after accommodation, 40 rats were randomly selected and divided into the following groups; healthy rats receiving 50 µl of phosphate-buffered saline (PBS) intratracheally (C group); sensitized rats receiving 50 µl of PBS intratracheally (S group); sensitized rats receiving 50 µl of PBS intratracheally containing 3×10^5^ C-kitˉ cells (S+C-kit^-^ group), and sensitized rats receiving 50 µl of PBS intratracheally containing 3×105 C-kit^+^ cells (S+C-kit^+^ group). The remaining rats were used for extraction of C-kit^+^ and C-kit^-^ cells.

***Induction of asthma***

The asthma model of the rat was established according to the previously used method (29, 30). Rats were sensitized by injection of 1 mg ovalbumin (OVA; Sigma-Aldrich, USA) mixed with 200 mg aluminum hydroxide (Sigma, Chemical Ltd, UK) dissolved in saline intraperitoneally on the first and 8^th^ days. From the 14^th^ day, animals were kept in a suitable whole-body inhalation exposure chamber with dimensions of 30×20×20 cm^3^ and exposed to 4% OVA aerosol produced by a nebulizer (CX3; Omron Co., Netherlands) for 5 min daily for 18±1 days (3 rats per each inhalation). The control group was challenged with normal saline instead of OVA. On the 33^rd^ day, PBS, PBS containing C-kitˉ and C-kit^+^ cells were injected into the trachea through a cervical incision in S, S+C-kit^-^, and S+C-kit^+^ groups, respectively. All animals were humanely euthanized two weeks later by cervical dislocation after a high-dose injection of xylazine and ketamine. 

***Magnetic-activated cell sorting (MACS)***

To isolate bone marrow-derived C-kit^+^ cells, rats were humanely euthanized by cervical dislocation after high-dose injection of xylazine and ketamine. Femurs were completely removed and medullary cells flushed out by PBS solution containing 2% fetal bovine serum (FBS; Gibco). The mononuclear cells were isolated by Ficoll (Sigma-Aldrich) gradient centrifugation at 400×g for 20 min. For the MACS procedure, harvested marrow mononuclear cells were incubated with 1% FBS for 30 min at 4 °C. After that, cells were exposed to anti-human C-kit microbead (Catalog no. 130-091-224; Miltenyi Biotec) and cells passed through the LS column (Miltenyi Biotec) to isolate the c-kit^+^ and c-kit^- ^cells (31).

***Cell labeling***

Both c-Kit^+^ and c-Kit^-^ cells were labeled using 20 µM Cell TrackerTM CM-Dil at 37 °C for 40 min followed by three-time PBS washes (32). In this study, 50 µl PBS containing 3×105 of positive and negative C-kit cells were used for injection per rat. 

***Flow cytometric analysis of systemic CD4*** ^+^ ***and CD8*** ^+^ ***lymphocytes***

The percentage of systemic CD4^+^ and CD8^+^ lymphocytes was measured before and after C-kit cell injection. For this purpose, blood cells were diluted with PBS solution (1:1) and mononuclear cells were isolated using Ficoll solution as above-mentioned. Then, cells were incubated with FITC-conjugated mouse anti-rat CD4 (eBioscience) and CD8 (eBioscience) for 30 min at 4 °C. Finally, cells were analyzed using the BD FACSCalibur flow cytometry system and FlowJo software (ver. 7.6.1).

***Western blotting***

The right lungs of each group were homogenized using lysis buffer and then the samples were centrifuged at 4 °C for 10 min at 10,000 rpm. The supernatant containing protein was determined by Bradford’s method. Equal amounts of protein were resolved by 10% sodium dodecyl sulfate-polyacrylamide gel electrophoresis (SDS-PAGE) and transferred to Polyvinylidene fluoride (PVDF) membranes (Millipore, Billerica, MA, USA). The membranes were washed with TBST buffer and blocked with 5% skimmed milk for 1 hr. After that, membranes were incubated with primary antibodies anti-β-actin (Catalog No: sc-47778), ERK 1/2 (Catalog No: sc-292838), p-ERK 1/2 (Catalog No: sc-16981-R), NF-ƙB p65 (Catalog No: ab16636) at 4 °C for 16–18 hr. Finally, the membrane was washed with TBST buffer and detected with an HRP-conjugated secondary antibody (Catalog No: sc-2357. The bands were visualized by enhanced chemiluminescence (ECL advanced reagents kit, Product Booklet, RPN2135).

***Immunofluorescence imaging (IF)***

Fourteen days after injection of Dil-labeled cells, 5-6 μm thick cryo-sections were prepared from lung tissues. The samples were incubated with anti-cytokeratin 19 antibody for 1 hr. After three-time PBS washes, the samples were exposed to an appropriate FITC-labeled secondary antibody for 1 hr at room temperature. The nuclei were stained using DAPI (4’,6-diamidino-2-phenylindol). Here, we monitored the existence of Dil+/cytokeratin-19+ cells (dual red/green stained cells), indicating the differentiation of transplanted cells into the pneumocyte-like cells (33). 

***Data analysis***

Results were displayed as mean±SEM and analyzed by one-way ANOVA with Tukey–Kramer post hoc test. Statistical P-values less than 0.05 were considered significant.

## Results

***Transplanted C-Kit*** ^+^ ***cells were engrafted into pulmonary tissue and differentiated into epithelial cells***

**Figure 1:**
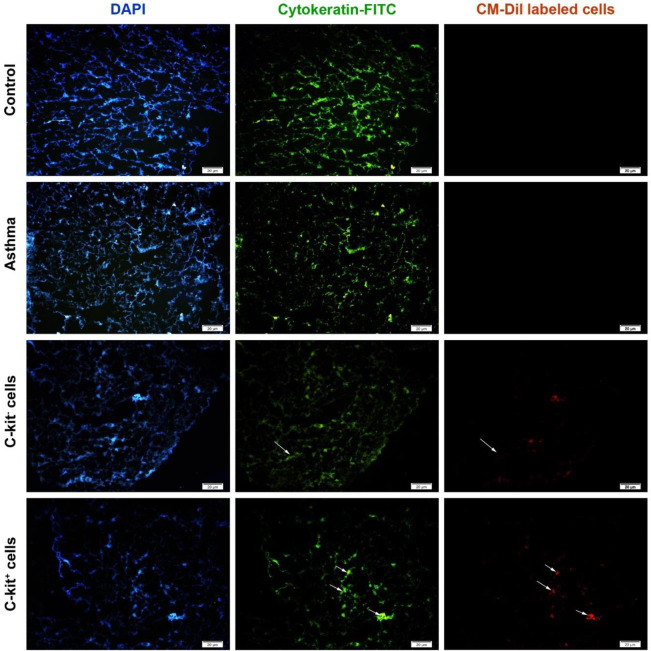
IF imaging of pulmonary tissue after intratracheal administration of C-kit cells. IF imaging revealed the existence of c-Kit labeled cells inside pulmonary niche shown by red-colored appearance. In the C-Kit^+^ group, the cells showed the potency to locate on the bronchial epithelium and through the alveolar niche. The number of labeled c-Kit- cells was low compared with C-Kit^+^ cells. C (control group), S (sensitized animals with ovalbumin), S+ckit^- ^(sensitized animals received c-kit- cells), S+ckit^+^ (sensitized animals received c-kit^+^ cells)

To check whether intra-tracheal administration of labeled C-kit^-^ and C-Kit^+^ cells can lead to successful homing into the pulmonary tissues, we performed an IF analysis. Data showed red-colored C-kit- and C-Kit^+^ cells inside the lung parenchymal 14 days after intratracheal administration. In S + C-kit^-^ and S + C-Kit^+^ groups, these cells were randomly distributed. It seems that the number of labeled cells was higher in the S+C-Kit^+^ group compared with the S+C-kit^-^ group. These data showed that intratracheal administration of C-kit^-^ and C-Kit^+^ cells led to successful recruitment into the pulmonary niche after 14 days in a rat model of asthma. We also performed an IF analysis based on Cytokeratin-19 staining. This factor is a typical pneumocytes protein marker. We found that C-kit cells had the potential to express Cytokeratin-19 fourteen days after transplantation into the rat model of asthma. Based on our data, the number of green-colored cells (Double green/red-stained cells) was high in the S+C-Kit^+^ group compared with S+C-kit^-^ rats, showing the prominent trans-differentiation capacity of C-kit^+^ cells compared with the C-kit^-^ subsets. As expected, labeled cells were not detectable in either sensitized (S) or control (C) groups. These data showed that C-kit^+^ progenitor cells can acquire a pneumocyte-like phenotype inside the asthmatic niche which can help the host lung to restore the function of resident pneumocytes (Figure 1). 

***Administration of C-kit*** ^+^ ***cells changed systemic levels of CD4*** ^+^ ***and CD8*** ^+^ ***lymphocytes in sensitized rats***

**Figure 2:**
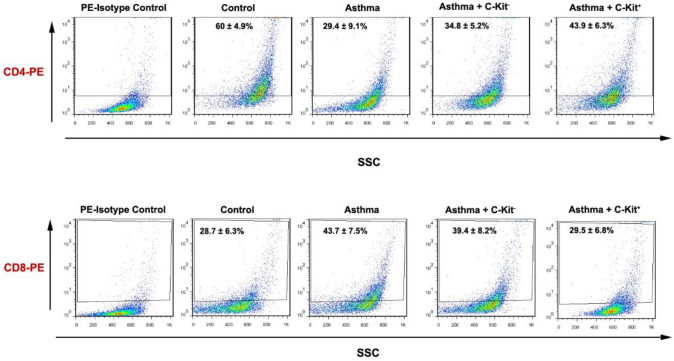
Flow cytometric analysis of rat peripheral mononuclear cells in different groups (A and B). The cells were stained with a panel of antibodies, including CD4^+^ and CD8^+^. The percentage of mononuclear cells expressing each marker is expressed as mean±SEM. C (control group), S (sensitized animals with ovalbumin), S+ckit- (sensitized animals received c-kit^-^ cells), S+ckit^+^ (sensitized animals received c-kit^+^ cells)

Flow cytometry results demonstrated that the percentage of CD4^+^ cells in the sensitized group was decreased (60 ± 4.9 vs 29.4 ± 0.1%) as compared with group C. According to our data, transplantation of C-kit^-^ and C-Kit^+^ cells via intra-tracheal route increased the systemic number of CD4^+^ cells in comparison with the asthmatic profile (34.8 ± 5.2 and 43.9 ± 6.3 vs 29.4 ± 0.1%, respectively). These changes were more evident in sensitized rats that received C-Kit^+^ cells (*P*<0.001). In contrast to dynamic changes of CD4^+^ cells, the blood levels of CD8^+ ^cells were diminished after the onset of asthmatic changes compared with the control rats (28.7 ± 6.3 vs 43.7 ± 7.5%, *P*<0.01). The transplantation of both C-kit^-^ and C-Kit^+^ cells reduced the abnormal elevation of CD8^+^ cells almost to near-to-control levels (39.4 ± 8.2 and 29.5 ± 6.5 vs 43.7 ± 7.5%, respectively). These data showed that transplantation of C-Kit^+^ cells in sensitized rats can modulate the abnormal lymphocyte counts. Although C-kit^-^ can alter the number of lymphocyte subsets under asthmatic conditions, these changes were less in sensitized rats that received C-Kit^+^ cells (*P*<0.05, Figure 2). 

***C-kit*** ^+^ ***cells suppressed ERK/NF-*** ƙ***B activity in sensitized rats***

**Figure 3:**
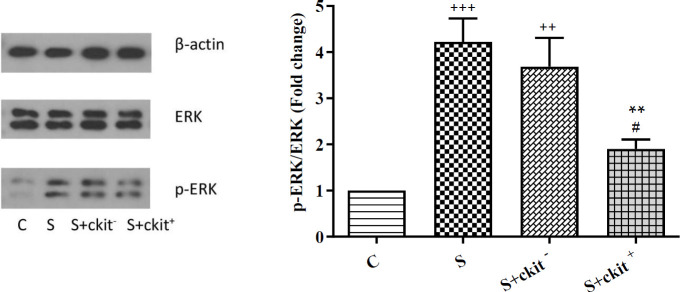
Protein level ratios of p-ERK/ERK in the lung tissues of C (control group), S (sensitized animals with ovalbumin), S+c-kit^- ^(sensitized animals received c-kit^-^ cells), and S+ckit^+^ (sensitized animals received c-kit+ cells) groups. Statistical differences between different groups vs control: +++; *P*<0.001, ++; *P*<0.01. Statistical differences between S+ ckit^+^ vs group S: **; *P*<0.01. Statistical differences between S+ckit^+^ and S+ckit^-^ groups: #; *P*<0.05. Bars represent the mean±SEM

Proteomic analysis revealed alteration of factors related to the ERK/NF-ƙB signaling pathway. Data showed an increased p-ERK/ERK ratio in sensitized rats (S) compared with the control (C) group (*P*<0.001). We noted that administration of C-kit^+^ but not C-Kit^-^ decreased abnormal phosphorylation of ERK effector compared with the sensitized group (*P*<0.05). Despite reduction of ERK phosphorylation in S+C-Kit^-^ and S+C-Kit^+^ groups, the p-ERK/ERK ratio was higher compared with the normal condition (Figure 3). 

**Figure 4:**
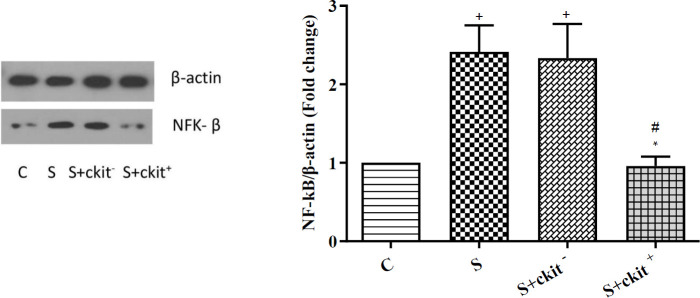
Protein level of NF-ƙB in the lung tissues of C (control group), S (sensitized animals with ovalbumin), S+ckit^-^ (sensitized animals received c-kit^-^ cells), and S+ckit^+^ (sensitized animals received c-kit+ cells). Statistical differences between different groups vs control: +; *P*<0.05. Statistical differences between S+ckit^+^ vs group S: *; *P*<0.05. Statistical differences between S+ckit+ and S+ckit- groups: #; *P*<0.05. Bars represent the mean±SEM

Along with these changes, it was notified that promotion of asthmatic conditions can alter protein levels of NF-ƙB inside the lungs. Consistent with the phosphorylation of ERK, the levels of NF-ƙB were increased in the lungs of the S group compared with the control rats (*P*<0.05). We showed that administration of C-Kit^+^ cells can reduce synthesis of NF-ƙB compared with the S group (*P*<0.05). Unlike the therapeutic effects of C-Kit^+^ cells, C-Kit^-^ cells did not change NF-ƙB compared with the S group (Figure 4). These data showed that promotion of asthmatic changes can alter specific signaling transduction pathways such as ERK/NF-ƙB axis which can lead to the inflammatory response. The application of specific progenitor types such as C-Kit^+^ cells reduced protein synthesis of NF-ƙB and diminished the p-ERK/ERK ratio, leading to suppression of inflammatory response in asthmatic lungs.

## Discussion

The global increase of asthma and the absence of completely effective modalities increase the need to invent new therapeutic approaches. Previously, the existence of special progenitor types such as C-Kit^+^ cells has been documented in the pulmonary tissue. However, the exact role of these cells in the dynamic healing of asthmatic lungs is lacking (34, 35). Some studies highlighted that pulmonary C-Kit^+^ cells can participate in tissue homeostasis and reconstitution (36-40). In the current experiment, the possible effect of intratracheally administered C-Kit^+^ and C-Kit- cells was investigated on the ERK/NF-ƙB pathway in a sensitized rat model. We also monitored the potency of C-Kit^+^ and C-Kit^-^ cells in the alteration of systemic CD4^+^ and CD8^+^ lymphocytes. Whether transplanted C-Kit^+^and C-Kit- cells can restore the asthmatic lung function was studied via monitoring differentiation toward cytokeratin-expressing cells (Cytokeratin-19). 

Here, we found that the number of CD4^+^ lymphocytes was declined during the asthmatic changes and intratracheal administration of C-Kit^+^ cells can, if not completely, restore the CD4^+^ lymphocyte numbers to near-to-normal levels. These effects were less in the sensitized group that received C-Kit- cells. In contrast to the changes of CD4^+^ lymphocyte number, the percent of CD8+ lymphocytes was increased after the onset of asthmatic changes. The transplantation of C-Kit^+^ cells, but not C-Kit^-^ cells, in sensitized rats decreased the abnormal increase of CD8+ lymphocytes to the control levels. It was suggested that increased CD8^+^ cytotoxic T lymphocytes and reduction of CD4^+^ lymphocytes are closely associated with airway hyperreactivity and asthmatic pathologies (11, 41-43). According to our data, the changes of CD4^+^ and CD8^+^ lymphocytes in asthmatic rats can attenuate airway inflammation and hyperresponsiveness (44). Consistent with our data, previous studies have shown that transplantation of C-Kit^+^ cells improved Th1/2 imbalance, pathological features, and secretion alterations of pro-inflammatory mediators, leading to the suppression of pathological remodeling in asthmatic lungs (35, 45, 46). Murine pulmonary C-Kit^+^ cells are eligible to restore lung function by reducing the release of Th2 pro-inflammatory cytokines IL-4, -5, and -13, coincided with an elevation of type 2 macrophage IL-10 (35). The findings of the current study comply with previous studies, suggesting the immunomodulatory effect of C-Kit^+^ cells in in vivo conditions by promotion of phenotype shifting in macrophages from a proinflammatory state to an anti-inflammatory condition (47). 

We also noted that progression of the asthmatic condition can lead to promotion of NF-ƙB and p-ERK/ERK signaling axes. Data showed that pulmonary NF-ƙB and p-ERK/ERK protein levels were increased in S and S+ C-Kit^-^ groups compared with control and sensitized rats that received C-Kit^+^ cells. It was suggested that MAPK/NF-ƙB signal pathways could adjust cellular processes like proliferation, differentiation, apoptosis, and production of immunomodulatory and pro-inflammatory mediators in airways (48, 49). Elevated expression of NF-ƙB can be associated with enhanced p-ERK/ERK, leading to pathological changes (50). It has to be proposed that activation of NF-ƙB/MAPK regulates maturation of dendritic cells (51) and differentiation of Th2 cells (52). Activation of the NF-ƙB inflammatory signaling pathway after MAPK activation might be responsible for the development of airway inflammation (11). In support of this notion, some researchers have suggested different treatments to improve OVA-induced airway inflammation via inhibiting MAPK/NF-κB axis (53-56). Since the levels of phosphorylated ERK1/2 and p38 MAPK in asthmatic cases correlate to the severity of airway disease (57, 58), inhibition of MAPK signal pathways was suggested as a new strategy for the treatment of airway inflammation and asthma (50, 59, 60). It is noteworthy to mention that C-Kit^+^ cells can regulate the immune system activity during asthmatic changes via alteration signaling cascades such as the MAPK/NF-κB pathway. Whether juxtacrine or paracrine activity of C-Kit^+^ cells participates in the modulation of MAPK/NF-κB axis needs further investigation. Kajstura and colleagues reported that injection of human lung C-Kit^+^ cells adjacent to the damaged areas in female mice promoted lung healing (36). 

## Conclusion

In the present study, we showed the orientation of transplanted C-Kit^+^ cells toward pneumocyte-like cells via monitoring the increase of Cytokeratin-19. Consistent with previous reports, C-Kit^+ ^cells can differentiate into different lineages to restore the activity of injured tissues. These data showed that reconstitution of injured pneumocytes during asthmatic changes can be one of the therapeutic potentials of C-Kit^+^ cells. 

## Authors’ Contributions

FM and RK Designed the study; RR Extracted C-kit^+^ and C-kit^-^ cells; FM, MA, and HH Contributed to acquisition of data; FM and RK Analyzed the data; FM Wrote the manuscript and RK edited the paper. All authors read and approved the final manuscript.

## Funding

This study was approved and supported equally by grants from the Stem cell research center of Tabriz University of Medical Sciences and Council for Development of Stem Cell Sciences and Technologies (No:11.35730-1398.8.17). 

## Conflicts of Interest

The authors declare that no conflict of interest exists.
